# Carbon dioxide laparoscopic technology: research hotspots and innovations in pneumoperitoneum detection

**DOI:** 10.4103/mgr.MEDGASRES-D-25-00206

**Published:** 2026-03-26

**Authors:** Jiajun He, Xinmei Pan

**Affiliations:** 1 Key Laboratory for Quality Evaluation of Active Devices, Tianjin Center for Medical Devices Evaluation and Inspection, Tianjin, China; 2 The International Peace Maternity & Child Health Hospital of China Welfare Institute (IPMCH), International Peace Maternity and Child Health Hospital, Shanghai Jiao Tong University School of Medicine, Shanghai, China

**Keywords:** adhesions, carbon dioxide, complications, gas embolism, hypercapnia, insufflator, laparoscopy, literature visualization analysis, medical gases, pain, pneumoperitoneum

## Abstract

As laparoscopic technology evolves rapidly, the safe management and precise detection of carbon dioxide (CO_2_) pneumoperitoneum have become the core link to ensure surgical safety. Herein, we attempt to systematically explore the research hotspots and innovative technologies in CO_2_ pneumoperitoneum detection through bibliometric analysis, patent mining and clinical trial data integration. A visual analysis was performed on relevant 80 articles retrieved from the Web of Science Core Collection. (1) Keyword analysis revealed that current research focuses on laparoscopic cholecystectomy, with major complications including postoperative pain, adhesion formation, and gas embolism. (2) Literature co-citation analysis showed that gas pretreatment technology significantly reduces hypothermia and postoperative pain. Relevant international guidelines emphasize the necessity of dynamic end-tidal carbon dioxide monitoring, while the mechanisms linking pneumoperitoneal pressure to cardiovascular and respiratory systems remain the focus of pathophysiological research. (3) Recent studies over the past 5 years have indicated that pneumoperitoneum pressure optimization has emerged as a central research focus. High-pressure pneumoperitoneum significantly increases intracranial pressure, and different surgical approaches variably impact acid-base balance. However, the effects of heated and humidified insufflation gas on postoperative inflammatory responses require further validation. (4) Patent analysis revealed that China’s patent-authorized technologies in this field are mainly pneumoperitoneum pressure constant control, gas temperature and humidity regulation, and intelligent monitoring equipment, among which the multi-channel constant-pressure insufflator and integrated humidification pipeline system have become the highlights of innovative technologies. (5) Clinical trial registration protocol analysis revealed that current cutting-edge clinical research focuses on three key areas in robotic surgery: ultra-low pressure pneumoperitoneum management, exploration of novel gas mixture ratios, and intelligent monitoring technologies. (6) The complications of CO_2_ pneumoperitoneum involve multi-system interactions: cardiovascular compression can affect hemodynamic stability, the respiratory system is vulnerable to the threat of hypercapnia, and prolonged duration of pneumoperitoneum may induce gastrointestinal dysmotility. (7) In terms of technological innovations, CO_2_ laparoscopic insufflator, intelligent monitoring tools (e.g., ultrasound assessment of intracranial pressure) and anesthesia management protocols (e.g., pharmacological suppression of hemodynamic fluctuations) have gradually shown their clinical potential, but the clinical benefits of gas temperature and humidity control remain controversial. To conclude, this paper reveals the current research status, hotspots and trends in the field of CO_2_ laparoscopic pneumoperitoneum detection, which provides a reference for the development of precision and safety in laparoscopic surgery.

## Graphical abstract

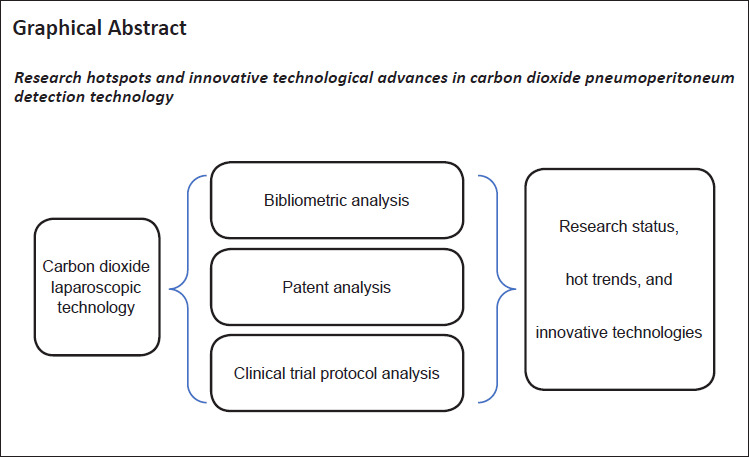

## Introduction

**Figure 1:**
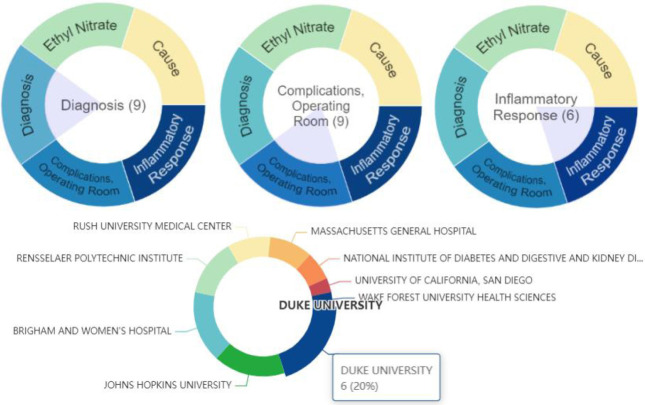
NIH-funded research projects on pneumoperitoneum. (A) Distribution of NIH-funded pneumoperitoneum studies. A search for “pneumoperitoneum” in the NIH RePORTER database (https://reporter.nih.gov/) identified 31 funded projects. The top three research fields are: diagnosis (9 projects), intraoperative complications (9 projects), and inflammatory responses (6 projects). (B) Institutional distribution of NIH-funded pneumoperitoneum studies. Duke University is the top-funded institution with the highest number of research projects (6 projects).

Pneumoperitoneum is a pathological condition in which free gas is present in the abdominal cavity. Normally, the human abdominal cavity is free of gas. When gas enters the abdominal cavity and accumulates due to various causes, pneumoperitoneum is developed.^1^ Pneumoperitoneum is triggered by gastrointestinal perforation, abdominal trauma, and intra-abdominal infections.^2^,^3^,^4^ Certain medical operations may also lead to pneumoperitoneum. For example, in laparoscopic surgery, carbon dioxide (CO_2_) needs to be injected into the abdominal cavity to form a pneumoperitoneum, facilitating surgical operations, but if the procedure is not performed properly, the gas may escape to other parts of the abdominal cavity, or the gas is not fully expelled after surgery, both of which may cause pneumoperitoneum.^5^ In addition, when gastroenteroscopy or enema is performed, improper procedures may also lead to gastrointestinal perforation, thereby causing pneumoperitoneum.^6^,^7^ In recent years, the U.S. National Institutes of Health is also concerned about pneumoperitoneum-related research. According to the report from the U.S. NIH RePORTER, there are 31 NIH-funded projects related to pneumoperitoneum, mainly focusing on the diagnosis of pneumoperitoneum, intraoperative complications and inflammatory response-related research. Among these, Duke University has the maximum number of research projects related to pneumoperitoneum (six projects), highlighting international attention to this field (**Figure 1**). Therefore, investigations into CO_2_ pneumoperitoneum detection have significant clinical implications and practical application value.

Laparoscopic surgery, as an important technique in modern surgery, has been widely used in clinical practice due to its significant advantages, such as less trauma, faster recovery, and less pain.^8^,^9^,^10^ CO_2_ has become the gas of choice for establishing pneumoperitoneum in laparoscopic surgery because of its colorless, odorless, nonflammable, relatively safe, and inexpensive properties.^11^,^12^ During laparoscopic surgery, insufflation of CO_2_ into the abdominal cavity induces pneumoperitoneum, which provides sufficient space and a good field of vision for surgical procedures.^13^

However, the establishment and maintenance of CO_2_ pneumoperitoneum is not without risk. On the one hand, changes in pneumoperitoneum pressure may affect the patient’s respiratory and circulatory systems to varying degrees.^14^,^15^ Excessive pneumoperitoneum pressure may lead to the elevation of the diaphragm, damage pulmonary ventilation, increase airway pressure, and cause alterations in respiratory mechanics. Meanwhile, it may also compress the inferior vena cava, leading to a decrease in the volume of returned blood and affecting the stability of the cardiovascular system. On the other hand, CO_2_ may leak and induce complications, such as subcutaneous emphysema and gas embolism, which may even endanger the patient’s life in severe cases.^16^,^17^,^18^ In addition, the quality of the pneumoperitoneum, such as the uniformity and stability of the pneumoperitoneum, also directly affects the surgical difficulty and effectiveness.^19^ As laparoscopic surgery continues to develop and become more popular, the complexity and difficulty of procedures have gradually increased, leading to higher demands for CO_2_ pneumoperitoneum.^20^ Therefore, how to accurately and real-time detect the relevant parameters of CO_2_ pneumoperitoneum to ensure the safety, stability, and effectiveness of the pneumoperitoneum has become an important issue of concern to be solved in laparoscopic surgery.

This study aimed to systematically sort out the current research hotspots related to CO_2_ laparoscopic pneumoperitoneum detection through literature visualization analysis, and to introduce relevant innovative techniques, with the expectation of providing new insights into the development of pneumoperitoneum detection in CO_2_ laparoscopy, thereby further refining and enhancing the laparoscopic surgical technique.

## Methods

### Search strategy

The Web of Science Core Collection database was searched by the first author on March 26, 2025, with no limitation of publication date and literature timeframe. Search for article titles was used. A total of 80 articles were finally included (**Table 1**).

**Table 1:** Literature search formula related to CO_2_ laparoscopic technique for pneumoperitoneum detection in the Web of Science Core Collection

#	Search formula	Results
1	TI=(Laparoscop*)	96,495
2	TI=("Carbon Dioxide" OR CO_2_ OR Capnography OR Capnocytophaga)	202,672
3	TI=(Pneumoperitoneum)	2,028
4	#3 AND #2 AND #1	80

CO_2_: Carbon dioxide.

### Data analysis

Eighty articles retrieved from the Web of Science Core Collection database were exported to TXT format files and initially subjected to data deduplication screening. The data were then imported into VOSviewer_1.6.20 software for visual analysis to generate co-occurrence maps of keywords and co-cited references.^21^,^22^ Using the VOSviewer_1.6.20 software (VOSviewer, CA, San Francisco, USA), clustering analysis was performed on the keywords and co-cited reference units.^23^ The clustering results were represented by nodes and connecting lines in different colors, and each cluster represented a research topic or research direction. By observing the size, distribution, and interrelationships of the clusters, research hotspots and research trends in the field could be identified. The VOSviewer maps were exported and converted to Pajek-supported file formats (net, clu, and vec files). Pajek64 software (Croatian Academy of Sciences and Arts, Zagreb, Croatia) was subsequently used for image conversion to construct network maps of keywords and co-cited references.^24^,^25^

The clustering results of the keyword co-occurrence network could reveal the research themes and directions in this field. Each cluster represents a relatively independent research topic. By analyzing the content and size of the clusters, research hotspots and key areas in this field could be identified. The size of a keyword node reflects its frequency of occurrence. Larger nodes indicate that the corresponding keywords appear more frequently in the literature, representing core research topics in the field. The citation frequency of a publication serves as an important indicator of its academic influence. A higher citation frequency indicates that the literature holds significant reference value and impact in this field.^26^,^27^

### Patent analysis

In the WanFang database, patent retrieval was performed on March 26, 2025 with the following search formula: subject: (carbon dioxide) AND subject: (laparoscopy) AND subject: (pneumoperitoneum OR pneumoperitoneum). Publication date range was unrestricted.

### Clinical registry protocols

Clinical trial protocols related to CO_2_ laparoscopic technology for pneumoperitoneum detection were searched in ClinicalTrials.gov and the Chinese Clinical Trial Registry, to summarize the registration progress of clinical trials in this field. In the Chinese Clinical Trial Registry, the search terms “laparoscopy” was used for the registered title and “pneumoperitoneum” for the disease under study. Publication date range was unrestricted.

## Results

### Results of keyword analysis

**Figure 2:**
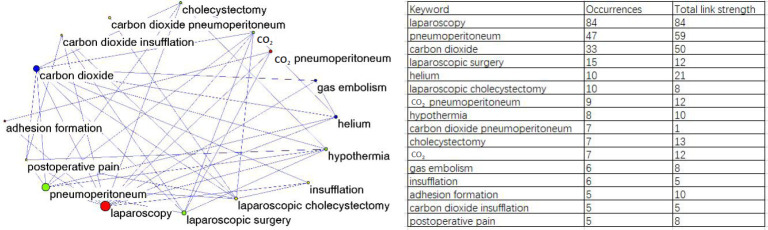
Co-occurrence network of high-frequency keywords in literature related to CO_2_ laparoscopy technique for pneumoperitoneum detection (Web of Science Core Collection database). The larger the total link strength (TLS) value, the more frequently the two keywords concurrently in the literature, indicating a closer association between them. Different colors represent different clusters, and the larger node represents the higher frequency of keyword occurrences. CO_2_: Carbon dioxide.

Keyword network mapping was performed using VOSviewer_1.6.20 and Pajek64 software, and a total of 16 keywords that appear more than 5 times were selected to generate co-occurrence network maps (**Figure 2**). The top 5 keyword frequencies were: 84 times for laparoscopy, 47 times for pneumoperitoneum, 33 times for carbon dioxide, 15 times for laparoscopic surgery, and 10 times for helium.

**Figure 3:**
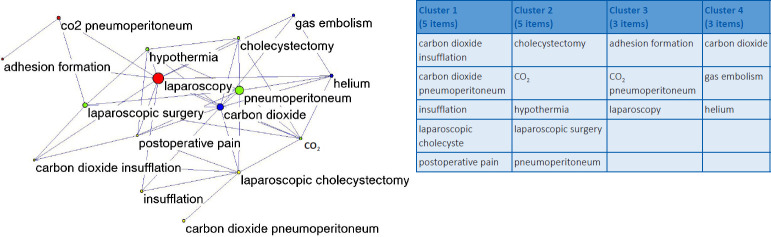
Keyword clustering network mapping of studies related to CO_2_ laparoscopy technique for pneumoperitoneum detection (Web of Science Core Collection database) Different colors in the left figure represent different clusters, and larger nodes represent higher keyword co-occurrences. The right figure shows the list of keyword clusters. CO_2_: Carbon dioxide.

The keyword clustering network mapping is shown in **Figure 3**. The keywords were divided into 4 clusters: cluster 1 (5 terms): carbon dioxide (CO_2_) insufflation, CO_2_ pneumoperitoneum, insufflation, laparoscopic cholecystectomy, postoperative pain; cluster 2 (5 terms): cholecystectomy, carbon dioxide, hypothermia, laparoscopy, pneumoperitoneum; cluster 3 (3 terms): adhesion formation, CO_2_ pneumoperitoneum, laparoscopy; cluster 4 (3 terms): CO_2_, gas embolism, helium. The results of keyword clustering indicate that the hot procedure in current research is CO_2_ laparoscopic cholecystectomy, and the main adverse effects are postoperative pain, adhesion formation, and gas embolism.

### Results of literature co-citation analysis

**Figure 4:**
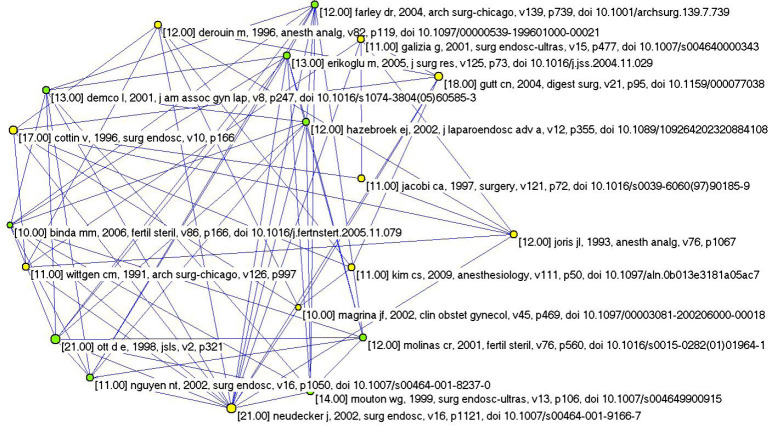
Co-citation network diagram of research literature related to CO_2_ laparoscopy technique for pneumoperitoneum detection (Web of Science Core Collection database). Different colors represent different clusters, and larger nodes represent the higher frequency of literature co-citations. The number in parentheses indicates the co-citation frequency. CO_2_: Carbon dioxide.

Using the VOSviewer_1.6.20 and Pajek64 software, literature co-citation network mapping was conducted on 19 articles with a co-citation frequency more than 10 times (**Figure 4** and **Table 2**).^28^,^29^,^30^,^31^,^32^,^33^,^34^,^35^,^36^,^37^,^38^,^39^,^40^,^41^,^42^,^43^,^44^,^45^,^46^

**Table 2:** Details on 19 cited publications related to carbon dioxide laparoscopic techniques for pneumoperitoneum detection (sorted by citation frequency)

First author	Publication year	Study type	Research topic	Citation	Total link strength
Ott et al.^28^	1998	Randomized controlled trial	Reducing laparoscopic surgery-induced hypothermia, postoperative pain, and recovery room length of stay through gas preconditioning using the Insuflow® Device	21	71
Neudecker et al.^29^	2002	Guidelines	Laparoscopic surgery for pneumoperitoneum	21	42
Gutt et al.^30^	2004	Review	Circulatory and respiratory complications of carbon dioxide insufflation	18	17
Cottin et al.^31^	1996	Case report	Gas embolism in laparoscopic surgery	17	29
Mouton et al.^32^	1999	Randomized controlled trial	Evaluating the benefits of humidified inflatable gases in laparoscopic surgery	14	48
Demco et al.^33^	2001	Randomized controlled trial	Effects of heated and humidified gases on awake patients undergoing laparoscopic surgery	13	50
Erikoglu et al.^34^	2005	Animal experiment	Electron microscopic changes in the peritoneum after cold and heated carbon dioxide pneumoperitoneum	13	49
Farley et al.^35^	2004	Randomized controlled trial	Warmed and humidified carbon dioxide insufflation vs. standard carbon dioxide insufflation in patients undergoing laparoscopic cholecystectomy	12	57
Hazebroek et al.^36^	2002	Animal experiment	Effect of temperature and humidity of carbon dioxide pneumoperitoneum on body temperature and peritoneal morphology	12	40
Derouin et al.^37^	1996	Observational study	Detection of gas embolism during laparoscopic cholecystectomy by transesophageal echocardiography	12	24
Molinas et al.^38^	2001	Animal experiment	Peritoneal mesothelial cell hypoxia during pneumoperitoneum is a cofactor in adhesion formation in a laparoscopic mouse model	12	15
Joris et al.^39^	1993	Observational study	Hemodynamic changes during laparoscopic cholecystectomy	12	12
Nguyen et al.^40^	2002	Randomized controlled trial	Effect of heated and humidified carbon dioxide on core temperature and postoperative pain	11	52
Kim et al.^41^	2009	Observational study	Venous air embolization during total laparoscopic hysterectomy	11	13
Galizia et al.^42^	2001	Randomized controlled trial	Hemodynamic and pulmonary changes during open, carbon dioxide pneumoperitoneum and abdominal lift cholecystectomy	11	9
Wittgen et al.^43^	1991	Observational study	Analysis of hemodynamic and ventilatory effects of laparoscopic cholecystectomy	11	7
Jacobi et al.^44^	1997	Animal experiment	Carbon dioxide pneumoperitoneum stimulates growth of malignant colonic cells	11	3
Binda et al.^45^	2006	Animal experiment	Effects of drying and temperature on adhesion formation in mice during laparoscopic surgery	10	37
Magrina et al.^46^	2002	Review	Complications of laparoscopic surgery	10	13

Of the 19 publications, 4 have been cited more than 15 times.^28^,^29^,^30^,^31^ A randomized controlled trial study by Ott et al.^28^ (cited 21 times) showed that pretreatment of laparoscopic gases by filtering, heating and rehydration using the Insuflow (Georgia BioMedical Inc.) is significantly superior to the standard feedstock gases currently in use, which is safe in terms of reducing or eliminating laparoscopy-induced hypothermia, shortening the recovery room length of stay, and reducing postoperative pain. A European guideline published by Neudecker et al.^29^ (cited 21 times) indicates that end-tidal carbon dioxide concentration must be monitored during laparoscopic surgery. The available data on closed (Veress needle) and open laparoscopic techniques do not allow investigators to predominantly favor the use of either technique. The use of a 2–5 mm rather than a 5–10 mm trocar slightly improves cosmetic results and postoperative pain. The lowest intra-abdominal pressure that permits adequate exposure of the operative area is recommended over the use of conventional pressures. In patients with limited cardiac, pulmonary, or renal function, an abdominal lift combined with low-pressure pneumoperitoneum may be an alternative. An abdominal lift device has no clinical advantage over low-pressure (5–7 mmHg; 1 mmHg = 0.133 kPa) pneumoperitoneum. In patients with cardiopulmonary disease, intraoperative and postoperative arterial blood gas monitoring is recommended. The clinical benefits of warmed, humidified insufflated gas are modest and contradictory. In all prolonged laparoscopic procedures, continuous intermittent pneumatic compression of the lower extremities is recommended intraoperatively. There are several treatment options available for the prevention of postoperative pain. Although all of them seem to reduce pain, the current data do not justify a general recommendation. A review by Gutt et al.^30^ (cited 18 times) concluded that the main pathophysiologic changes during CO_2_ pneumoperitoneum involve the cardiovascular system, which are mainly related to intra-abdominal pressure and the patient’s position on the operating table. Even elderly and weak patients tolerate these changes well, with no significant cardiovascular complications other than a slight increase in the incidence of arrhythmias. Significant pathophysiologic changes in the lungs, hypercapnia, hypoxemia, and barometric pressure surges may occur, but are rare due to the use of effective ventilation monitoring and techniques. Changes in splanchnic perfusion are proportional to the increase in intra-abdominal pressure and the duration of pneumoperitoneum. Successive organ dysfunctions are minor, transient, and do not affect the therapeutical outcome; therefore, moderate to low intra-abdominal pressures (< 12 mmHg) help limit these pathophysiological changes. Cottin et al.^31^ (cited 17 times) strongly emphasized the need for precise rules to prevent gas embolism during laparoscopic surgery and for close monitoring of cardiac rhythm during CO_2_ insufflation. Patients with a history of surgery should be considered at high risk.

There are 15 publications with 10–14 citations.^32^,^33^,^34^,^35^,^36^,^37^,^38^,^39^,^40^,^41^,^42^,^43^,^44^,^45^,^46^ A randomized controlled trial by Mouton et al.^32^ (cited 14 times) showed that the use of humidified inflatable gas can reduce pain after laparoscopic cholecystectomy, but the holding effect of humidified inflatable gas is not significant. Another randomized controlled trial by Demco et al.^33^ (cited 13 times) indicated that heated and humidified CO_2_ can improve patient’s tolerance to awake laparoscopic surgery while reducing the frequency and duration of shoulder pain. In an animal study by Erikoglu et al.^34^ (cited 13 times), heated and humidified CO_2_ led to fewer peritoneal alterations than dry-cooled CO_2_. Another animal study by Erikoglu et al.^34^ (cited 13 times) showed that heated and humidified CO_2_ resulted in fewer peritoneal changes than dry-cooled CO_2_. Therefore, this study concluded that heated, humidified CO_2_ is more suitable for pneumoperitoneum in laparoscopic surgery, especially in specific cases. A randomized controlled trial by Farley et al.^35^ (cited 12 times) showed that patients undergoing laparoscopic cholecystectomy with heated, humidified CO_2_ inflation could maintain a higher intraoperative core body temperature compared with those undergoing laparoscopic cholecystectomy with standard CO_2_ inflation. Dry-cooled CO_2_ insufflation may decrease body temperature during laparoscopic surgery, which has been indicated in an animal study by Hazebroek et al.^36^ (cited 12 times). Heating and humidifying the insufflated gas can prevent hypothermia. After CO_2_ insufflation, the peritoneal surface changes despite heating or humidification, as it does after gasless surgery. An observational study by Derouin et al.^37^ (cited 12 times) evaluated 16 patients [American Society of Anesthesiologists (ASA) physical status I–III] undergoing laparoscopic cholecystectomy and found that gas embolism was common during laparoscopic cholecystectomy but minimally caused the cardiopulmonary instability. Molinas et al.^38^ (cited 12 times) demonstrated the feasibility of a mouse model of intubation laparoscopy in an animal experiment, indicating a key role of mesothelial cell hypoxia in adhesion formation. An observational study by Joris et al.39 (cited 12 times) showed that laparoscopic cholecystectomy in the supine position leads to significant hemodynamic changes in healthy control group, especially when pneumoperitoneum is induced. Nguyen et al.^40^ (cited 11 times) conducted a randomized controlled trial addressing the use of external heating blankets combined with heated and humidified gases that could minimize intra-abdominal temperatures without altering core temperature or reducing postoperative pain. An observational study by Kim et al.^41^ (cited 11 times) revealed a 100% incidence of air embolism in patients undergoing total laparoscopic hysterectomy. Compared with alternative total laparoscopic hysterectomy, total laparoscopic hysterectomy has a higher grade of air embolism, especially when the round ligament is transected and the broad ligament is dissected. A randomized controlled trial by Galizia et al.^42^ (cited 11 times) showed that CO_2_ pneumoperitoneum can severely affect cardiopulmonary function. In high-risk patients, gasless laparoscopic surgery is preferred due to its reliability and the absence of cardiopulmonary changes. An observational study by Wittgen et al.^43^ (cited 11 times) revealed that careful intraoperative monitoring of CO_2_ uptake in arterial blood gases may be necessary in patients with chronic cardiopulmonary disease during laparoscopic cholecystectomy. Jacobi et al.^44^ (cited 11 times) showed that compared with helium and controls, CO_2_ insufflation promoted tumor growth in a rat model. An animal study by Binda et al.^45^ (cited 10 times) explored the effects of drying (not cooling) and supersaturation of pneumoperitoneum on adhesion formation and found that drying increased adhesions, whereas lowering body temperature decreased adhesions. Adhesions were minimized when humidified gas was used. The effect of drying is usually underestimated because of the counteracting effect with cooling. The concept of combining controlled intraperitoneal cooling with strict prevention of drying may be important for clinical prevention of adhesions. A review by Magrina et al.^46^ (cited 10 times) provides insight into the various complications of gynecologic laparoscopic surgery. Complications regarding CO_2_ laparoscopic technique for pneumoperitoneum detection primarily include subcutaneous emphysema, mediastinal emphysema, pneumothorax, gas embolism, hypercapnia, and postoperative shoulder pain.

### Results of hotspot analysis

From 2020 to 2025, a total of 11 original research articles related to the latest innovations in CO_2_ laparoscopic technique for pneumoperitoneum detection were found (**Table 3**).^47^,^48^,^49^,^50^,^51^,^52^,^53^,^54^,^55^,^56^,^57^ Current research focuses on the optimization of pneumoperitoneum pressure, the protection of organ function, and the innovation of anesthesia management methods. Future advancements should integrate intelligent monitoring technologies, personalized protocols, and novel gas applications to further enhance surgical safety and patient prognosis.

**Table 3:** Details on 11 original research publications related to carbon dioxide laparoscopic techniques for pneumoperitoneum detection in 2020–2025 (sorted by cited frequency)

Author	Publication year	Study design	Research topic	Research technology	Source title	Times cited, WoS Core
Yashwashi et al.^47^	2020	Non-randomized controlled trial	Observation of the effect of low- and high-pressure carbon dioxide pneumoperitoneum on intracranial pressure during laparoscopic cholecystectomy	Detection of intracranial pressure during laparoscopic cholecystectomy by measuring the optic nerve sheath diameter using ultrasonography.	*Surgical Endoscopy and Other Interventional Techniques*	13
Liu et al.^48^	2021	Randomized controlled trial	Observation of the effect of carbon dioxide pneumoperitoneum on acid-base balance during laparoscopic inguinal hernia repair	During primary inguinal hernia repair, blood gas analysis and dynamic transcutaneous carbon dioxide changes were used to analyze the acid-base balance.	*Hernia*	8
Ogawa et al.^49^	2020	Single-arm trial	Predictive performance of isoproterenol target-controlled infusion during robotic-assisted laparoscopic prostatectomy with carbon dioxide pneumoperitoneum on the head-down position	Head-down position and carbon dioxide pneumoperitoneum detection required for robot-assisted laparoscopic prostatectomy	*Journal of Anesthesia*	3
Gunusen et al.^50^	2022	Randomized controlled trial	Effects of carbon dioxide pneumoperitoneum at different temperatures and humidities on hemodynamic and respiratory parameters and postoperative pain during gynecologic laparoscopic surgery	Recommendations for heating and humidifying carbon dioxide during laparoscopic surgery to prevent postoperative pain and hypothermia	*Asian Journal of Surgery*	2
Badarudeen et al.^51^	2021	Animal experiment	Observation of the effect of the duration of carbon dioxide pneumoperitoneum on gastrointestinal dysmotility after laparoscopic surgery	Observation of the independent effect of carbon dioxide pneumoperitoneum on gastrointestinal dysmotility or postoperative ileal stasis	*Indian Journal of Surgery*	2
Bolat et al.^52^	2022	Non-randomized controlled trial	Should we use low-pressure carbon dioxide pneumoperitoneum during laparoscopic cholecystectomy?	Pneumoperitoneum using low- and high-pressure carbon dioxide in laparoscopic surgery	*Annali Italiani Di Chirurgia*	1
Morais et al.^53^	2020	Animal experiment	Does carbon dioxide pneumoperitoneum during laparoscopic surgery affect collagen deposition in abdominal surgical wounds?	Determining whether carbon dioxide pneumoperitoneum interferes with collagen deposition in rat surgical wounds by histomorphometric analysis	*Acta Cirurgica Brasileira*	1
Peng et al.^54^	2024	Randomized controlled trial	Application of different carbon dioxide pneumoperitoneum pressures in laparoscopic pyeloplasty in infants with ureteropelvic junction obstruction	Laparoscopic pyeloplasty with carbon dioxide pneumoperitoneum	*Frontiers in Pediatrics*	0
Verguts et al.^55^	2025	Randomized controlled trial	Does the addition of nitrous oxide and oxygen to carbon dioxide pneumoperitoneum for laparoscopic surgery reduce pain?	Can an alternative insufflation gas mixture consisting of 86% carbon dioxide, 10% nitrous oxide, and 4% oxygen used for pneumoperitoneum in gynecologic laparoscopic surgery reduce pain?	*Bjog-An International Journal of Obstetrics And Gynaecology*	0
Atasever et al.^56^	2023	Observational study	Effect of lateral 45° head-down position and carbon dioxide pneumoperitoneum on optic nerve sheath diameter in patients undergoing laparoscopic transperitoneal nephrectomy: a prospective observational study	Assessment of the effect of carbon dioxide pneumoperitoneum on optic nerve sheath diameter	*Journal of Laparoendoscopic & Advanced Surgical Techniques*	0
Bagle et al.^57^	2022	Randomized controlled trial	Effects of clonidine *vs*. magnesium sulfate on the hemodynamic response to carbon dioxide pneumoperitoneum in patients undergoing laparoscopic surgery	Intravenous administration of 1 μg/kg clonidine or 30 mg/kg magnesium sulfate before pneumoperitoneum is effective in suppressing pressurized response during laparoscopic surgery	*Journal of Clinical and Diagnostic Research*	0

A non-randomized controlled trial by Yashwashi et al.^47^ (cited 13 times) showed that high-pressure pneumoperitoneum resulted in a significant increase in intracranial pressure compared with low-pressure pneumoperitoneum during laparoscopic cholecystectomy. A randomized controlled trial by Liu et al.^48^ (cited 8 times) compared the effect of CO_2_ pneumoperitoneum on acid-base balance during transabdominal pre-peritoneal (TAPP) and total extraperitoneal (TEP) hernia repair and found that TAPP and TEP yielded favorable results in the treatment of inguinal hernia. Although no serious complications occur, TEP surgery may cause more severe CO_2_ accumulation and more rapid acidosis than TAPP surgery. A single-arm trial by Ogawaet al.^49^ (cited 3 times) observed the predictive performance of isoproterenol-targeted infusion during robotic-assisted laparoscopic prostatectomy with head-down CO_2_ pneumoperitoneum, confirming that the predictive effect was acceptable. A randomized controlled trial by Gunusen et al.^50^ (cited 2 times) revealed that both standard CO_2_ and heated and humidified CO_2_ had no effect on hemodynamic and respiratory parameters in Healthy controls. Only a higher core body temperature and inflammatory response were found in the heated and humidified CO_2_ group. In an animal experiment by Badarudeen et al.^51^ (cited 2 times), in addition to the central inhibitory effects of anesthetics and CO_2_, the duration of CO_2_ pneumoperitoneum did lead to gastrointestinal dysmotility after a prolonged surgical procedure. A non-randomized controlled trial by Bolat et al.^52^ (cited 1 time) showed no significant difference in clinical or laboratory outcomes between the use of low- and high-pressure CO_2_ pneumoperitoneum in laparoscopic surgery. Therefore, a pressure level that is easy to use and has a low complication rate is preferred. Morais et al.^53^ (cited 1 time) showed in an animal experiment that at 5 mmHg, CO_2_ pneumoperitoneum did not interfere with collagen deposition in surgical wounds of the abdominal wall in rats. A randomized controlled trial by Peng et al.^54^ showed that laparoscopic pyeloplasty for infants with ureteropelvic junction obstruction results in a more pronounced physiologic response, including acid-base alterations, stress response, and elevated inflammatory cytokines. Verguts et al.^55^ found that an alternative insufflation mixture used for pneumoperitoneum in gynecologic laparoscopic surgery consisting of 86% CO_2_, 10% N_2_O, and 4% O_2_ did not reduce postoperative pain when compared with standard insufflation gas of 100% CO_2_. An observational study by Atasever et al.^56^ utilized ultrasound optic nerve sheath diameter to assess the degree of intracranial pressure elevation in patients undergoing laparoscopic transperitoneal nephrectomy in both side-lying and 45° head-down positions. Additionally, the study evaluated the effect of CO_2_ pneumoperitoneum on optic nerve sheath diameter. The differences increased progressively with higher CO_2_ pneumoperitoneum levels and showed a positive correlation with surgical duration. At 24 postoperative hours, the difference returned to the baseline level. A randomized controlled trial by Bagle et al.^57^ showed that intravenous administration of either 1 μg/kg clonidine or 30 mg/kg magnesium sulfate prior to pneumoperitoneum effectively suppressed the pressurized response during laparoscopic surgery.

### Patent analysis results

A total of 58 Chinese patent items were retrieved, including 12 authorized patents. Ruijin Hospital of Shanghai Jiaotong University School of Medicine had three authorized patents, which is the Chinese institution with the most authorized patents in this research field. Most of the authorized patents focused on laparoscopic CO_2_ insufflators, humidifier, abdominal wall opening supporter, CO_2_ pneumoperitoneum pipeline, among which there are more patents in the category of laparoscopic CO_2_ insufflators (**Table 4**).

**Table 4:** Details on 12 granted Chinese patents related to CO_2_ laparoscopic technique for pneumoperitoneum detection in WanFang database (sorted by relevance)

Patent number	Patent	Patentee	Inventor (Designer)	Date of application	Public date
CN211511759U	Laparoscopic CO_2_ pneumoperitoneum heating and humidifying device	Second Affiliated Hospital of Nanchang University	Chenni Guo	2019-12-16	2020-09-18
CN114176666B	A CO_2_ pneumoperitoneum pipeline with constant pressure, constant temperature and humidifying functions	Ruijin Hospital of Shanghai Jiao Tong University School of Medicine	Xinjun Sheng, Yanchao Zhao, Haoxuan Zheng, Wei Cai, Jianwei Lin	2021-12-28	2023-11-14
CN222549321U	A laparoscopic incision sealing device with self-contained perforator trocars	Nanjing Drum Tower Hospital	Shengxian Fan, Gang Chen	2024-02-26	2025-03-04
CN217488722U	A laparoscopic insufflator	Shouxian Hospital of Traditional Chinese Medicine	Na Wang	2022-06-02	2022-09-27
CN221060864U	A laparoscopic smoke exhaust device	Qin Huang	Qin Huang, Meiying Pan, Shanling Wang	2023-10-08	2024-06-04
CN220001717U	Modified laparoscopy	Yanhui Zhao	Yanhui Zhao, Xiufen Li	2023-05-09	2023-11-14
CN219538257U	A laparoscopic integrated smoke and mist evacuation system	First Affiliated Hospital of Henan University of Science and Technology	Bo Li	2023-03-28	2023-08-18
CN219048426U	A laparoscopic fluorescence system	GuangDong ANESTHESIA Medical investment and Development Co., Ltd.	Yimeng Ou, Yadong Zhang, Shunjun Chen, Wanli Liu	2022-11-09	2023-05-23
CN114176684B	An abdominal wall opening supporter for laparoscopic surgery	Xiangya Hospital of Central South University	Xiwu Ouyang, Lemeng Feng, Ning Bai, Lei Yao, Xinying Li	2022-01-18	2023-03-21
CN215534649U	Multi-channel single-port laparoscopic surgical device	Ecan Medical Co., Ltd.	Quangen Liao	2021-06-30	2022-01-18
CN215230768U	An insufflator with heating function	Recrown	Jian Zhu, Qian Xue, Haiguang Wang	2021-05-24	2021-12-21
CN210249936U	Multi-functional laparoscopic surgical assist system	Ruijin Hospital of Shanghai Jiao Tong University School of Medicine	Wei Cai, Ming Tian, Pinhao Lu, Zheyu Yang, Yeding Yu	2019-05-31	2020-04-07

CO_2_: Carbon dioxide.

### Results of clinical registration protocols

Two registered clinical trial protocols were retrieved, both of which were observational studies. Among them, the latest study protocol was registered in 2022 by Nanfang Hospital of Southern Medical University, titled “Evaluation of the effect of Trendelenburg position on intracranial pressure in patients undergoing laparoscopic pneumoperitoneum surgery by ultrasonic” (ChiCTR2200065603; **Table 5**).

**Table 5:** Clinical trial protocols regarding carbon dioxide laparoscopic technology for pneumoperitoneum detection registered in the Chinese Clinical Trial Registry

Registration ID	Title/Institution	Study type	Registration date
ChiCTR2200065603	Evaluation of the effect of Trendelenburg position on intracranial pressure in patients undergoing laparoscopic pneumoperitoneum surgery by ultrasonic measurement of optic nerve sheath diameter: a prospective observational study	Observational study	2022-11-09
	Nanfang Hospital of Southern Medical University		
ChiCTR1900022320	The effect of pneumoperitoneum in the steep Trendelenburg position on cerebral oxygenation and cerebrovascular autoregulation during robotic-assisted urology surgery	Observational study	2019-04-04
	Anesthesia and Surgery Center of the First Medical Center of the Chinese PLA General Hospital		

In the ClincialTrial.gov (https://clinicaltrials.gov/), using the search formula: Pneumoperitoneum | Other terms: “Carbon Dioxide” OR CO_2_ OR Capnography OR Capnocytophaga | Laparoscopy OR Laparoscopies OR Laparoscopic, 50 registration entries were retrieved, as shown in **Table 6**. These protocols cover a wide range of types of laparoscopic surgeries, from prostate cancer surgery to liver surgery to colorectal surgery, and their research objectives include evaluating the efficacy of different surgical approaches, comparing the effects of different pneumoperitoneum pressures, and assessing the effects of breathing exercises on surgical patients. These studies are important for improving the safety and efficacy of laparoscopic surgery. The top 10 protocols, ranked by initial registration date, are presented in Table 6, which focus on the following types of laparoscopic surgery: (i) Robot-assisted radical prostatectomy (NCT06784986): to evaluate the impact of the Airseal system with ultra-low pneumoperitoneum in patients undergoing robot-assisted radical prostatectomy. (ii) Minimally invasive major liver surgery: to compare high and low peritoneal effusion compression for liver parenchyma resection in minimally invasive major liver surgery (NCT06770803). (iii) Laparoscopic cholecystectomy: to assess the effect of dexmedetomidine in reducing the MAC-BAR value of sevoflurane during pneumoperitoneum (NCT06575179); to assess the effect of respiratory exercises on abdominal distension in patients undergoing laparoscopic cholecystectomy (NCT06069557); and to compare the changes in intraoperative hemodynamic parameters and arterial blood gases at two different values of abdominal pressure (NCT05992636). (iv) Laparoscopic colorectal surgery: to evaluate the effect of low pressure combined with warm and humidified CO_2_ insufflation in laparoscopic colorectal surgery (NCT05934981). These protocols cover surgeries at different sites such as the prostate, liver, gallbladder and colorectal, as well as robotic-assisted and conventional laparoscopic surgical approaches. These studies cover multiple aspects, including surgical outcomes, pneumoperitoneal pressure, respiratory movement and hemodynamics. One of the most recent studies is a randomized controlled trial by the Brazilian Institute of Robotic Surgery registered in 2025, titled “Impact of Using the Airseal System with Ultra-low Pneumoperitoneum in Patients Undergoing Robot-assisted Radical Prostatectomy: a Prospective, Comparative, Randomized Clinical Study” (NCT06784986).

**Table 6:** Clinical trial protocols registered in ClinicalTrial.gov (https://clinicaltrials.gov/) regarding carbon dioxide laparoscopic technology for pneumoperitoneum detection (sorted by first registration date)

NCT number	Study title	Enrollment (*n*)	Study status	Study type	Conditions	First posted
NCT06784986	Impact of using the airseal system with ultra-low pneumoperitoneum in patients undergoing robot-assisted radical prostatectomy: a prospective, comparative, randomized clinical study	200	Recruiting	Interventional	Prostate Carcinoma|Prostate Neoplasm|Pain Intensity Assessment|Quality of Life (QOL)	2025-01-20
NCT06770803	High *vs*. low pneumoperitoneum pressure for parenchymal transection in minimally invasive major liver surgery	132	Not_Yet_Recruiting	Interventional	Liver Surgery|Minimal Invasive Surgery|Major Liver Resection	2025-01-13
NCT06575179	Dexmedetomidine reduces sevoflurane MAC-BAR during pneumoperitoneum	90	Recruiting	Interventional	Laparoscopic Cholecystectomy	2024-08-28
NCT06338865	Short title: standard *vs*. lower pressure pneumoperitoneum	294	Recruiting	Interventional	Laparoscopic Surgery	2024-04-01
NCT06224868	Comparison of the effects of different PEEP values with usg on optic nerve sheath diameter	45	Not_Yet_Recruiting	Interventional	Cholecystitis	2024-01-25
NCT06069557	The effect of respiratory exercises on abdominal distension in laparoscopic cholecystectomy patients	156	Active_Not_Recruiting	Interventional	Elective Laparoscopic Cholecystectomy	2023-10-06
NCT05992636	Different insufflation flows and effects on cerebral oxygenation in laparoscopic cholecystectomy	69	Completed	Observational	Pneumoperitoneum	2023-08-15
NCT05934981	Laparoscopic colorectal surgery using low-pressure combined with warm and humidified carbon dioxide insufflation	148	Recruiting	Interventional	Colorectal Surgery|Benign or Malignant Rectal or Colon Tumors	2023-07-07
NCT05558826	Pressure analysis of trendelenburg position effect on indices from arterial pressure	100	Unknown	Observational	Anesthesia|Fluid and Electrolyte Imbalance|Hemodynamic Instability	2022-09-28
NCT05367557	Comparison of intraoperative hemodynamic parameters and arterial-blood gas changes at two different pneumoperitoneal pressure values	20	Completed	Observational	Pneumoperitoneum	2022-05-10

## Discussion

### Literature analysis

A total of 16 keywords that appeared more than 5 times throughout the text were selected to generate a co-occurrence network diagram, and the top 5 keywords by frequencies were laparoscopy, pneumoperitoneum, CO_2_, laparoscopic surgery, and helium. The keywords were divided into four categories, with the hotspot procedure being CO_2_ laparoscopic cholecystectomy, and the main adverse effects being postoperative pain, adhesion formation, and gas embolism.

A total of 19 articles with more than 10 citations were selected to construct a literature network mapping, and the most highly cited articles focused on the effect of Insuflow device in gas preconditioning and guidelines for monitoring laparoscopic surgery. The other articles centered on pathophysiological changes during pneumoperitoneum, related complications, and the effect of heated and humidified gas. These 19 articles focused on issues related to pneumoperitoneum in CO_2_ laparoscopic surgery, with the common problems and hotspots centering on the effects of pneumoperitoneum on the patient’s cardiovascular system, respiratory system, and body temperature, as well as gas treatment approach (heated and humidified gas), and the prevention and control of complications (gas embolism, adhesion formation, subcutaneous emphysema, and hypercapnia).

From 2020 to 2025, there were 11 original research articles, with hotspots focusing on the optimization of pneumoperitoneum pressure, organ function protection, and innovations in anesthesia management methods. A total of 58 Chinese patents were retrieved, with 12 authorized patents. Among them, Ruijin Hospital of Shanghai Jiao Tong University School of Medicine had the highest number of authorized patents, most related to laparoscopic CO_2_ insufflators. Analysis of clinical registration protocols revealed that the Chinese Clinical Trial Registry included two observational studies, while the ClinicalTrial.gov listed 50 studies covering various laparoscopic surgeries, involving research on surgical outcomes, pneumoperitoneum pressure, and other aspects.

### Issues related carbon dioxide laparoscopic techniques for pneumoperitoneum detection

#### Issues related to intraoperative use of carbon dioxide

(1) Cardiovascular system: the pneumoperitoneum pressure and the patient’s position can cause pathophysiologic changes in the cardiovascular system. Elevated intra-abdominal pressure may compress the inferior vena cava, leading to a decrease in venous return and affecting the stability of the cardiovascular system. For example, Gutt et al.^30^ indicated a slight increase in the incidence of arrhythmias, even though it was mostly tolerated by elderly and frail patients. Joris et al.^39^ found that induction of pneumoperitoneum in the supine position during laparoscopic cholecystectomy resulted in significant hemodynamic changes in patients.

(2) Respiratory system: It can lead to important pulmonary pathophysiologic changes such as hypercapnia, hypoxemia, and pneumoperitoneum pressure surges. Elevated pneumoperitoneal pressure cause the elevation of the diaphragm, which interferes with pulmonary ventilation and increases airway pressure. Kim et al.^41^ showed a 100% incidence of air embolism in patients undergoing total laparoscopic hysterectomy. Derouin et al.^37^ noted that gas embolism is common during laparoscopic cholecystectomy, although the resulting cardiopulmonary instability is minimal.

(3) Heated and humidified gas: Farley et al.^35^ and Hazebroek et al.^36^ showed that hypothermia can be prevented by heating and humidifying the insufflated gas, but the peritoneal surface remains altered after CO_2_ insufflation.

(4) Gastrointestinal dysfunction: The animal experiment by Badarudeen et al.^51^ showed that, in addition to the central inhibitory effects of anesthetics and CO_2_, the duration of pneumoperitoneum can indeed lead to gastrointestinal dysfunction after prolonged surgery.

#### What are the complications of carbon dioxide pneumoperitoneum?

(1) Gas embolism: It is a serious complication that can lead to cardiopulmonary dysfunction or is even life-threatening. Cottin et al.^31^ emphasized the need for precise preventive rules during laparoscopic surgery and close monitoring of cardiac rhythms during CO_2_ insufflation. Patients who have undergone previous surgery should be considered to be in a high-risk group.

(2) Adhesion formation: It is related to factors such as peritoneal mesothelial cell hypoxia caused by CO_2_ pneumoperitoneum. Molinas et al.^38^ demonstrated through animal experiments that mesothelial cell hypoxia plays a key role in adhesion formation. Binda et al.^45^ found that dryness increases adhesions, whereas lowering the body temperature reduces adhesions. The use of humidified gas can minimize adhesions to the greatest extent.

(3) Hypercapnia: During laparoscopic surgery, CO_2_ pneumoperitoneum increases the partial pressure of CO_2_ in the blood, leading to respiratory acidosis. This process may jeopardize the patient, especially in specific groups such as patients with cardiopulmonary dysfunction. The increased intra-abdominal pressure leads to hemodynamic changes and has a direct effect on lung function, further increasing the risk of hypercapnia.^30^,^46^

(4) Subcutaneous emphysema, mediastinal emphysema, and pneumothorax: CO_2_ may leak, leading to complications such as subcutaneous emphysema, mediastinal emphysema, and pneumothorax. Magrina et al.^46^ conducted an in-depth review of the various types of complications during gynecologic laparoscopic surgery. CO_2_ leakage may cause subcutaneous emphysema. It refers to the condition where air enters the subcutaneous tissue and causes localized swelling. This condition usually occurs when gas enters the tissue and cannot be released in time, potentially leading to patient discomfort and, in extreme cases, respiratory distress. Mediastinal emphysema refers to the entry of air into the mediastinal space, which can lead to compression of the lungs, heart, and other vital organs, thus affecting respiratory and circulatory functions. Mediastinal emphysema typically occurs when gas diffuses into the mediastinum through anatomical structures, such as rupture or injury to the bronchus, trachea, or thoracic cavity, which may occur during surgery due to overinflation of the gas or other operative errors, especially during thoracic surgery. Pneumothorax is a serious complication where air enters the pleural cavity and makes the lung collapse. This condition usually occurs during surgery or after trauma, where damage to the chest wall or high internal pressure causes the entry of air into the pleural cavity. Pneumothorax often requires emergency treatment to ensure that the patient’s ability to breathe is uncompromised.

(5) Postoperative shoulder pain: This is one of the common complications of CO_2_ laparoscopic technique for pneumoperitoneum detection. Postoperative shoulder pain is mainly caused by CO_2_ pneumoperitoneum. Residual CO_2_ in the abdominal cavity at the end of the operation stimulates the diaphragm and causes shoulder pain. Sudden change in intra-abdominal pressure makes CO_2_ enter the subdiaphragmatic region through diaphragmatic orifices or tiny fissures, inducing a neurologic response. Heated and humidified CO_2_, which has been mentioned in several publications, can reduce the frequency and duration of shoulder pain, indirectly suggesting the association between postoperative shoulder pain and CO_2_ pneumoperitoneum.^46^

#### How to avoid hypercapnia

To avoid hypercapnia during CO_2_ laparoscopic surgery, a multifaceted approach can be taken. Gutt et al.^30^ showed that pneumoperitoneum is controlled by maintaining a moderately low intra-abdominal pressure of less than 12 mmHg to minimize CO_2_ uptake. Careful selection of the appropriate laparoscopic technique is essential to minimize leakage. The lowest pressure that provides adequate surgical field exposure should be used during surgery to prevent excessive pressures that result in large amounts of CO_2_ entering the bloodstream. In terms of respiratory management, effective monitoring should be used to monitor respiratory parameters, and ventilation strategies should be adjusted according to the results, with appropriate ventilation modes to ensure alveolar ventilation. Preoperative assessment of cardiopulmonary function is essential to formulating individualized plans for high-risk patients, and arterial blood gas analysis should be continuously performed during surgery. During gas processing, heated and humidified carbon dioxide gas is used, with the injection speed and volume controlled to ensure the airtightness of the pneumoperitoneum system. Postoperative care is essential, including continuous monitoring of respiration and blood gases, encouraging patients to perform deep breathing and coughing exercises, and providing respiratory support therapy when necessary. These comprehensive measures effectively prevent hypercapnia and ensure surgical safety for patients.

#### What are the effects of prolonged pneumoperitoneum

In CO_2_ laparoscopic techniques for pneumoperitoneum detection, prolonged pneumoperitoneum can have multiple adverse effects. From the perspective of organ function, the animal experimental results by Badarudeen et al.^51^ showed that, in addition to the central inhibitory effects of anesthetics and CO_2_, the duration of CO_2_ pneumoperitoneum did lead to gastrointestinal dysmotility after prolonged surgery, suggesting that prolonged pneumoperitoneum affects the function of the gastrointestinal tract, which may trigger problems such as slowed intestinal peristalsis. In terms of respiration and acid-base balance, prolonged pneumoperitoneum may lead to increased CO_2_ accumulation, triggering acid-base imbalance. Liu et al.^48^ compared transabdominal preperitoneal (TAPP) and total extraperitoneal (TEP) inguinal hernia repair and found that, although no serious complications occur, pneumoperitoneum in TEP surgery may lead to more severe CO_2_ accumulation and more rapid acidosis. Prolonged pneumoperitoneum may elevate such risks. In terms of its effect on patient’s physiologic status, prolonged pneumoperitoneum may also increase the probability of other adverse effects. For example, Yashwashi et al.^47^ indicated that high-pressure pneumoperitoneum leads to a significant increase in intracranial pressure, and prolonged pneumoperitoneum may further exacerbate this elevation, posing a potential risk to patients. During prolonged pneumoperitoneum, patients may be subjected to the maintenance of specific surgical positions and sustained intra-abdominal pressure, thereby increasing patient discomfort and impeding postoperative recovery. For example, in laparoscopic surgery, prolonged CO_2_ pneumoperitoneum may affect hemodynamic and respiratory parameters and increase cardiopulmonary burden in patients.

Overall, prolonged pneumoperitoneum duration in in CO_2_ laparoscopic procedures will adversely affect various aspects, such as gastrointestinal function, respiratory acid-base balance, intracranial pressure and overall physiological state. It is essential to reasonably control the duration of pneumoperitoneum in clinical practice to ensure surgical safety and postoperative recovery.

#### Technical points of carbon dioxide pneumoperitoneum management

At the technical level, precise control of pneumoperitoneum pressure is crucial. High-pressure pneumoperitoneum can significantly increase intracranial pressure. Surgical teams should select the appropriate pressure according to patient’s specific conditions and the type of surgery, and prioritize the pressure level that is easy to operate with a low complication rate. For the management of insufflated gas, heating and humidification can prevent hypothermia and reduce postoperative pain to a certain extent, but the clinical benefits are contradictory, which need to be applied after comprehensive consideration. In addition, high attention should be paid to the protection of organ function. Excessive duration of pneumoperitoneum can trigger a series of issues, such as gastrointestinal dysmotility, CO_2_ accumulation and acidosis, impacting the patients’ organ function. Thereby, the duration of pneumoperitoneum needs to be reasonably controlled.

In terms of technical management, it is essential to strengthen intelligent monitoring, assessing changes in intracranial pressure with the help of techniques such as ultrasound measurement of optic nerve sheath diameter, and continuously tracking patient’s physiological status. Individualized pneumoperitoneum management protocols are preferred, such as adopting the alternative method of abdominal lifting combined with low-pressure pneumoperitoneum for patients with cardiopulmonary function limitation. Meanwhile, emphasis on the innovation of anesthesia management, such as intravenous drug administration before pneumoperitoneum to inhibit the pressurized response, can comprehensively improve the safety of surgery and patient prognosis.

#### How to choose the appropriate carbon dioxide flow

In CO_2_ laparoscopic technique, it is very critical to choose the appropriate CO_2_ flow based on the type of surgery and the need. Here are specific suggestions based on the research team’s clinical experience, for reference only:

(1) Low-flow settings: Low-flow (e.g., 1–2 L/min) CO_2_ not only provides adequate surgical field of view, but also reduces the impact on the cardiovascular and respiratory systems. For example, one study found that during laparoscopic cholecystectomy, the use of low-pressure (10 mmHg) and low-flow CO_2_ settings significantly reduced postoperative shoulder pain and other discomforts.^58^

(2) High-pressure and high-flow settings: In specific situations, such as obese patients or procedures requiring complex maneuvers, high-flow (4– 6 L/min) and high-pressure (14–15 mmHg) CO_2_ may be necessary to generate adequate peritoneal insufflation and operating space. However, this setting requires enhanced real-time patient monitoring.^59^

(3) Innovative technologies and devices: AirSeal® is a novel pneumoperitoneum maintenance system that can maintain a stable pneumoperitoneum at lower flow rates and pressures through continuous smoke removal and CO_2_ recycle while reducing intraoperative smoke accumulation and impact on the surgical field. Studies have shown that this system has promising applications in a wide range of laparoscopic surgeries and can further optimize the selection of CO_2_ flow rates.^60^,^61^,^62^ Compared with cold-dry CO_2_, heated and humidified CO_2_ at low-flow settings significantly alleviates postoperative discomfort, maintains normothermia, and reduces surgical complications. In addition, heated and humidified CO_2_ can reduce pneumoperitoneum-induced tissue dryness and adhesions and improve postoperative recovery.^63^,^64^,^65^

Although many studies have been conducted on the selection of CO_2_ flow rates, there are still many issues to be further investigated. For example, there may be significant differences in the tolerance to CO_2_ flow rates among different types of surgeries and patient groups. Future studies could be more carefully categorized to provide more personalized flow selection criteria. In addition, integrating artificial intelligence and big data analytics to develop intelligent CO_2_ flow regulation devices is a promising future direction.

#### Correct use of insufflators for laparoscopy

The more common clinical insufflator is a kind of medical equipment used in laparoscopy, primarily designed to expand the field of vision and improve the accuracy and safety of the procedure. The laparoscopic insufflator can inflate the patient’s abdominal cavity or luminal organs by injecting an appropriate amount of air or CO_2_, thereby expanding the laparoscopic field of view. This facilitates observation and diagnosis by physicians while preventing damage or irritation to the visceral or luminal walls caused by the laparoscope. It also reduces patient pain and discomfort, ensuring the accuracy and safety of the laparoscopic examination. It should be noted that CO_2_ absorption may lead to a series of hemodynamic changes and alterations in acid-base balance, which need to be carefully managed intraoperatively.^66^,^67^

Initial parameters are set according to the patient’s condition and type of surgery. Recommendations from experts are as follows: for most surgeries in adults, the initial pneumoperitoneum pressure can be set at 1–15 mmHg, and the flow rate can be initially set according to the surgical needs. For example, the initial flow rate can be set at 1–3 L/min for precise surgeries, and when establishing the pneumoperitoneum, the flow rate can be set at 5–10 L/min for general surgeries. Specific procedures are: during a diagnostic or therapeutic laparoscopy procedure, the intra-abdominal CO_2_ injection was performed and the device measures the intra-abdominal pressure at short intervals and continuously compares the nominal pressure with the actual abdominal pressure. The function of the device is to maintain the nominal pressure. Through an automatic venting system, the intra-abdominal overpressure can be reduced to the preset nominal pressure. If the nominal flow rate is set too low, the nominal pressure will not be reached. During gas injection, it should be noted that CO_2_ is absorbed (endosmosis). This indicates that the body has absorbed part of the injected CO_2_. In extreme cases, excessively high CO_2_ concentrations in the blood or respiratory system may be fatal. To minimize this risk, careful and close monitoring of the patient’s vital signs should be throughout the gas injection to ensure that the patient is breathing well. Adequate breathing can help avoid or eliminate the consequences of CO_2_. High pressure or high flow rates increase CO_2_ uptake. The operator can fully expand the abdominal cavity with a pressure of 10–15 mmHg. Pressure exceeding 15 mmHg is used only in exceptional cases but increase the risk of endosmosis (insufflation-related complications). Intra-abdominal pressure should never exceed 30 mmHg.

In the context of modern technology, the development of smart insufflators offers the possibility of personalized and safer intraoperative management. New devices not only monitor pneumoperitoneum pressure and flow in real time, but also include smoke exclusion features to increase the clarity of surgical field and reduce the risk of contamination.^68^,^69^

### Limitations

To accurately locate the research hotspot literature, title search is adopted to retrieve a relatively small number of publications in the field (only 80 articles), but all of them have a certain degree of academic representativeness. Only one database, Web of Science Core Collection, was searched. While not exhaustive, the search results provide relatively precise identification of research hotspots.

### Conclusions and prospects

The article systematically sorts out the research hotspots of pneumoperitoneum detection techniques in CO_2_ laparoscopic techniques through literature visualization and analysis. The current hotspot procedure is CO_2_ laparoscopic cholecystectomy, and the main adverse effects include postoperative pain, adhesion formation and gas embolism. During the use of CO_2_ laparoscopy, there are cardiovascular, respiratory and other systemic problems and multiple complications. The article also discusses the avoidance of hypercapnia, the effect of pneumoperitoneum time, technical management, flow rate selection, and the use of insufflators. Future research must overcome three challenges: first, to develop an intelligent pneumoperitoneum system that integrates real-time monitoring of multiple parameters, such as pressure, temperature, humidity, and biomarkers; second, to establish a model for the dynamic regulation of pneumoperitoneum based on patients’ individual characteristics, such as cardiorespiratory function and type of surgery; and third, to explore the application scenarios of alternative gases, such as helium, and to optimize the gas-mixing strategy in order to balance surgical vision and physiological disturbances.

## Data Availability

*Not applicable.*
